# Risk prediction models for short-term mortality in ICU stroke patients: a systematic review and meta-analysis

**DOI:** 10.3389/fneur.2025.1623645

**Published:** 2025-07-16

**Authors:** Jiali Zhang, Yijie Fu, Yan Liu, TianHeng Liu, Yue Deng, LiFei Dai, Tianmin Zhu, Hui Li

**Affiliations:** ^1^School of Preclinical Medicine and School of Nursing, Chengdu University, Chengdu, Sichuan, China; ^2^Clinical Medical College and Affiliated Hospital, Chengdu University, Chengdu, Sichuan, China; ^3^Health and Rehabilitation College, Chengdu University of Traditional Chinese Medicine, Chengdu, Sichuan, China

**Keywords:** stroke, mortality, risk, prediction models, ICU

## Abstract

**Objectives:**

This study aims to systematically review and evaluate risk prediction models for short-term mortality in ICU stroke patients, thereby providing scientific evidence to inform future model development and clinical application.

**Methods:**

We searched the Cochrane Library, EMBASE, PubMed, and Web of Science for studies on prediction models for short-term mortality in ICU stroke patients, covering the period from January 2005 to January 2025. Data extracted included study characteristics and detailed information on the prediction models. The Risk of Bias and applicability of the models were evaluated using the Prediction Model Risk of Bias Assessment Tool (PROBAST). A meta-analysis was performed using a random-effects model in Stata 18.0, and heterogeneity across studies was assessed using the I^2^ statistic. Subgroup analyses were conducted based on stroke type, geographic region, and modeling approach. a sensitivity analysis performed to evaluate the robustness of the findings.

**Results:**

A total of 6,874 studies were retrieved, and 12 studies met the inclusion criteria, yielding 14 prediction models, as two studies included two models each that were extracted separately. Four models were externally validated. The reported area under the curve (AUC) values ranged from 0.761 to 0.977. Meta-analysis yielded a pooled AUC of 0.82 (95% CI: 0.80–0.85), indicating good discriminative ability of the models in predicting short-term mortality in ICU stroke patients. However, heterogeneity was high (I^2^ = 80.1%, *p* = 0.000). Subgroup analyses by stroke type, modeling approach, and geographical region revealed no statistically significant sources of heterogeneity. The PROBAST assessment shows that all models exhibit high risk of bias and low applicability. The most frequently reported predictors were Glasgow Coma Scale (GCS), white blood cell count (WBC), age, and blood glucose levels.

**Conclusion:**

This study shows that prediction models for short-term mortality in ICU stroke patients have good discriminatory performance. However, due to high bias risk and low applicability, their overall quality remains suboptimal. Important predictors such as GCS, WBC, age, and blood glucose levels should be included in future models. Future research should focus on prospective, multicenter, and externally validated studies guided by the PROBAST tool to improve clinical applicability and reliability.

**Systematic review registration:**

https://www.crd.york.ac.uk/PROSPERO/recorddashboard.

## Introduction

Stroke is a major global health concern and remains one of the leading causes of death and long-term disability worldwide (1). Each year, approximately 6 million people die due to stroke, accounting for more than 10% of global mortality (2). Although stroke incidence has stabilized and mortality rates have declined, the overall burden of stroke continues to increase, with a rising number of new cases, survivors, and stroke-related deaths reported over the past two decades (3). A substantial proportion of stroke patients require admission to the intensive care unit (ICU) for neurological monitoring or management of severe complications, with an estimated 10–30% considered critically ill (4). Moreover, the economic burden associated with stroke treatment and long-term care is substantial, with healthcare expenditures projected to reach approximately USD 1.84 trillion between 2012 and 2030 (5). Given these challenges, early identification of patients at high risk of mortality is crucial for guiding clinical decision-making and improving outcomes.

Risk prediction models have gained increasing prominence in both medical research and clinical practice. These models incorporate multiple predictive variables into statistical frameworks to estimate the likelihood of adverse clinical outcomes (6, 7). Early identification of high-risk patients through these models facilitates timely interventions to reduce mortality and complications. However, despite the increasing number of models developed to predict short-term mortality in ICU stroke patients, systematic evaluations of their methodological rigor, potential biases, and real-world applicability remain limited.

## Objective

This study aims to systematically review and evaluate existing risk prediction models for short-term mortality in ICU stroke patients, providing scientific evidence to inform the development of future high-quality models and their clinical application.

## Methods

### Design

This systematic review followed the CHecklist for critical Appraisal and data extraction for systematic Reviews of prediction Modeling Studies (CHARMS) checklist (8) and the PROBAST tool (9) for critical appraisal and risk of bias assessment. The protocol was prospectively registered in PROSPERO (CRD420251009136).

### Search strategy

A comprehensive search was conducted across multiple databases, including Cochrane, EMBASE, PubMed and Web of Science. The search strategies were tailored to each database. Core search terms included “stroke,” “intensive care unit,” “mortality,” “prediction model,” and “risk.” The full search strategies for all databases are provided in Supplementary Table S1. This systematic review sought to identify studies published between January 2005 and January 2025 that focus on risk prediction models for short-term mortality in stroke patients admitted to the ICU. The inclusion criteria were based on the Participants, Interventions, Comparisons, Outcomes, Timing, and Setting (PICOTS) framework, as outlined below:

P (Population): Stroke patients admitted to the ICU.

I (Intervention): Risk prediction models for short-term mortality.

C (Comparator): Not applicable.

O (Outcome): Short-term mortality.

T (Timing): During hospitalization.

S (Setting): Stroke patients in the ICU only.

### Inclusion and exclusion criteria

*Inclusion criteria:* (1) Studies involving hospitalized patients aged 16 years or older; (2) studies that developed or validated prediction models including at least two predictive variables; (3) studies in which the primary outcome was short-term mortality; (4) studies focusing on critically ill stroke patients admitted to the intensive care unit (ICU); (5) studies with observational designs, including prospective and retrospective cohort studies, as well as case–control studies, that developed or validated prediction models.

*Exclusion criteria*: (1) Studies without full-text availability; (2) conference abstracts, magazine articles, commentaries, opinion pieces, newsletters, and other forms of gray or secondary literature; (3) student dissertations; (4) articles containing erroneous data or methodological flaws; (5) non-English language publications.

### Study selection

Duplicate records were removed using NoteExpress. Two independent reviewers (Zhang Jiali and Li Hui) screened the titles and abstracts of retrieved studies based on the predefined inclusion and exclusion criteria. Any discrepancies were resolved through discussion or by consulting a third reviewer (Fu Yijie). After initial screening, the same two reviewers independently assessed the full texts of potentially eligible articles. Additionally, the reference lists of the included studies were manually searched to identify other relevant publications.

### Data extraction

Data extraction was performed by two independent reviewers (Zhang Jiali and Li Hui) using a pre-designed extraction table. The extracted data included basic details such as authorship, publication year, country of origin, study design, and data source. For the prediction models, we collected detailed information on modeling methods, validation types, performance metrics, handling of missing data, and the predictors included in the final model. In studies presenting multiple models, we focused on the model with the highest AUC value during the model development phase. This review does not specifically focus on stroke patients with certain underlying conditions, such as diabetes or hypertension, and the included studies did not stratify based on comorbidities. In case of discrepancies, a third reviewer (Fu Yijie) was consulted to reach consensus.

### Quality assessment

The risk of bias (ROB) and applicability of the prediction models in the included studies were assessed using the PROBAST tool. This tool evaluates four domains: participants, predictors, outcomes, and analysis, with the first three domains also assessing applicability. Each item is rated as “yes,” “probably yes,” “no,” “probably no,” or “no information.” A domain is considered to have a high risk of bias if at least one item is rated as “no” or “probably no.” If one or more domains are rated as unclear while the others are rated as low risk, the overall ROB is considered unclear. An overall low ROB requires all domains to be rated as low risk. Two independent reviewers (Zhang Jiali and Li Hui) performed the quality assessment using PROBAST. Any discrepancies were resolved by consulting a third reviewer (Fu Yijie).

### Statistical analysis

The meta-analysis was conducted using Stata software, version 18. Standard errors for the AUC values were automatically calculated by Stata during the meta-analysis process using the built-in meta-analysis commands. Given the high degree of heterogeneity observed across the included studies, a random-effects modelwas applied to the meta-analysis. To explore potential sources of heterogeneity, we conducted subgroup analyses based on stroke type, geographic region, and modeling approach. The discriminatory ability of the models was assessed using Area Under the Curve (AUC) from the Receiver Operating Characteristic (ROC) analysis. A higher AUC value (closer to 1.0) indicates better discriminatory power of the model. Additionally, a sensitivity analysis was performed by sequentially excluding individual studies to assess the robustness of the pooled AUC estimates.

## Results

### Selection process

**Figure 1:**
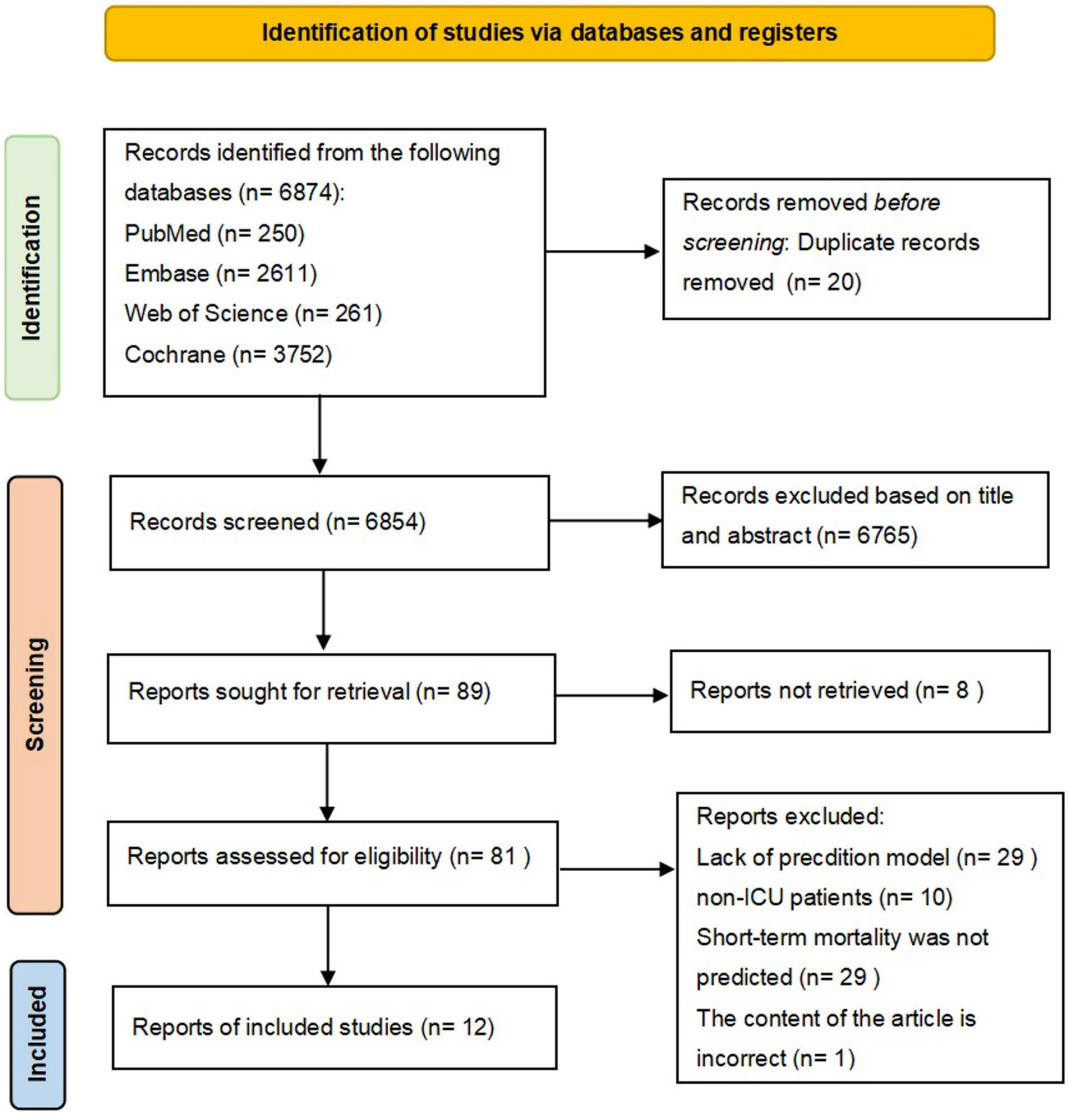
Preferred reporting items for systematic reviews and meta-analyses (PRISMA) flowchart of literature search and selection.

A comprehensive search across multiple databases (Cochrane, EMBASE, PubMed, and Web of Science) identified a total of 6,874 records. After removing duplicates (*n* = 20), 6,854 records were screened for title and abstract evaluation. Ultimately, 12 studies meeting the inclusion criteria were included in this review (10–21). Figure 1 presents the Preferred Reporting Items for Systematic Reviews and Meta-Analyses (PRISMA) 2020 Flow Diagram detailing the process of literature identification, screening, and selection.

### Study characteristics

This systematic review included 12 studies that investigated risk prediction models for short-term mortality in ICU stroke patients (10–21). These studies, published between 2022 and 2024, were conducted in the United States, the Netherlands, Brazil, and France. All studies employed a retrospective study design and derived data from existing databases, with most extracting data on the first day of ICU admission or within 24 h (10–13, 15, 19–21). However, four studies did not specify the timing of data extraction (14, 16–18). The total sample size across all studies was 45,939 participants, with individual sample sizes ranging from 236 to 16,592. The highest mortality rate, 41%, was observed in studies focusing on hemorrhagic stroke (HS) (13). Stroke diagnoses were primarily based on the International Classification of Diseases, Ninth and Tenth Revisions (ICD-9 and ICD-10) (10–12, 14–17, 20, 21). Detailed study characteristics are summarized in Table 1.

**Table 1:** Basic information.

First author	Publication Year	Country	Study design	Data source	Data extraction time	Total sample (total mortality rate)	Reference Standard for Stroke Definition	Stroke type	Age (Median/Mean, Years)	Outcome indicator (days)
Haoran Chen (10)	2024	America	Retrospective study	The MIMIC IV and III database	The first day of ICU admission	2,982 (23.6%)	ICD-9 and ICD-10	IS and HS	66.6 ± 14.99	30
Lingyan Fang (11)	2024	America	Retrospective study	The MIMIC-III database	The first day of ICU admission	2089 (−)	ICD-9 and ICD-10	IS	68.92 (57.33, 78.86)	28
Guangyong Jin (12)	2023	America	Retrospective study	The MIMIC IV database	The first day of ICU admission	1,259 (26.37%)	ICD-9 and ICD-10	IS	76.83 (70.93, 82.82)	28
Mariëlle K van Valburg (13)	2024	Dutch	Retrospective study	The Dutch National Intensive Care Evaluation database.	Within 24h after ICU admission	14,303 (IS: 27%, HS: 41%)	–	IS and HS	IS: 70 (59, 78) HS: 63 (51, 73)	30
Jian Huang (14)	2023	America	Retrospective study	The MIMIC IV database eICU-CRD	–	2,526 (19%)	ICD-9	IS and HS	71.2 (60.91, 81.6)	28
Forhan Bin Emdad (15)	2023	America	Retrospective study	The MIMIC-III database	Within 24h after ICU admission	757 (40.6%)	ICD-9	HS	Most are above 70 years	7
Jianyu Zou (16)	2022	America	Retrospective study	The MIMIC-III database	–	890 (−)	ICD-9	HS	Training: 71.00 (58.00, 81.00) Validation: 70.00 (59.00, 80.00)	30
Longyuan Gu (17)	2023	America	Retrospective study	The MIMIC-III database data collected at institution.	–	548 (31%)	ICD-9	HS	61 (50.75,71)	7
Pedro Kurtz (18)	2022	Brazil	Retrospective study	Electronic system from Brazilian hospitals	–	16,592 (8%)	–	IS and HS	70 (55, 81)	30
Qing Mei (19)	2024	French	Retrospective study	Public database	Some at admission or within 24 h	236 (28.81%)	–	HS	56 (46, 64)	30
Yuxin Wang (20)	2023	America	Retrospective study	The MIMIC IV database	Within 24 h after ICU admission	2,990 (7-day: 12.6%; 28-day: 19.6%)	ICD-9 and ICD-10	HS	–	7, 28
Xiao-Dan Li (21)	2022	America	Retrospective study	The MIMIC-III database	The first day of ICU admission	767 (25.0%)	ICD-9	–	70 (58, 80)	30, 180, 360

IS, Ischemic Stroke; HS, Hemorrhagic Stroke; ICD, International Classification of Diseases; the MIMIC III and IV database, the Medical Information Mart for Intensive Care (MIMIC) III and IV databases; eICU-CRD, eICU Collaborative Research Database; Study (13) developed two stroke-type–specific models: one for IS and one for HS; study (21) developed two models for HS: one predicting 7-day mortality and one predicting 28-day mortality; Only the 30-day prediction model from Xiao-Dan Li (22) was included in this review, The 180-day and 360-day models were excluded.

Table 2 presents the characteristics of the prediction models used in the included studies. The sample sizes used solely for model development varied considerably, ranging from a minimum of 341 to a maximum of 13,274. The modeling approaches primarily consisted of traditional statistical models and machine learning methods. Studies addressing missing data exclusively employed imputation methods (10–12, 14–16, 18–20), while some did not report their approach to handling missing data (13, 17, 21). All studies conducted internal validation; however, only four performed external validation (10, 14, 16, 17), with relatively limited external validation results. Various calibration methods were employed, with calibration curves and Brier scores being the most commonly used. The reported AUC values during model development ranged from 0.761 to 0.977. A total of 7 machine learning models were developed (10, 14–18, 20), with the Multilayer Perceptron (MLP) model achieving an AUC of 0.977 and the Random Forest (RF) model reaching 0.90 (17, 18). These machine learning models demonstrated relatively higher AUC values compared to traditional statistical models, such as logistic regression and LASSO regression, with the latter achieving an AUC of approximately 0.795 (19). The final presentation formats of the models included nomograms and web-based calculators (10–12, 16, 17, 19, 21). These characteristics of the prediction models are detailed in Table 2.

**Table 2:** The characteristics of the prediction models.

First author	Sample size/example			Modeling approach	Methods for handling missing data	Validation method	AUC			Calibration method	Presentation format
	Model building	Iinternal validation	Eexternal validation				MModel development	IInternal validation	Eexternal validation		
Haoran Chen (10)	2,386 (80%)	596 (20%)	2, 252	Explainable machine learning	Impute missing values	Internal validation External validation	0.88 ± 0.01	-	0.84 ± 0.01	Calibration curve Brier score	Nomogram
Lingyan Fang (11)	1,443	646	–	Binary logistic regression	Impute missing values	Internal validation	0.834 (0.810–0.859)	0.839 (0.804–0.874)	–	Calibration curve	Nomogram
Guangyong Jin (12)	894	365	-	Binary logistic regression	Impute missing values	Internal validation	0.809 (0.778, 0.841)	0.786 (0.737, 0.835)	–	Calibration curve	Nomogram
Mariëlle K van Valburg (13)	IS: 4005 HS: 2776	4,417 3,105		Logistic regressions	–	internal validation	is: 0.85 (0.84–0.87) ICH: 0.85 (0.83–0.86)	0.85 (0.84–0.87) 0.85 (0.83–0.86)	-	Calibration plots Brier scores	–
Jian Huang (14)	2031	495	1748	Interpretable machine learning	Impute missing values	Internal validation External validation	0.822	0.739	0.700	Calibration plots	–
Forhan Bin Emdad (15)	605	152	-	machine learning	impute missing values	internal validation	0.82	-	-	–	
Jianyu Zou (16)	Modeling + internal validation = 623		267	Machine learning	Multiple imputations	Internal validation External validation	0.772 (0.732–0.811)	0.778 (0.719–0.838)	–	Calibration curves The Hosmer–Lemeshow test	Nomogram
Longyuan Gu (17)	341		207	Machine learning methods	–	Internal validation External validation	0.977	0.913	–	Calibration curves	Web-based online calculator
Pedro Kurtz (18)	13,274 (80%)	3,318 (20%)	–	Machine learning regression	Multiple imputations	Internal validation	0.90	–	–	Calibration belts Brier score	–
Qing Mei (19)	–	–	–	Logistic regression	Multiple imputations	Internal validation	0.795 (0.731–0.858)	0.780	–	Calibration plot The Hosmer–Lemeshow test	Nomogram
Yuxin Wang (20)	2093 (70%)	897 (30%)/	–	Ensemble learning method	Multiple imputations	Internal validation	7 days:0.875 (0.842–0.908) 28 days:0.761 (0.712–0.809)	–	–	Calibration curve Brier Score	–
Xiao-Dan Li (21)	536	231	–	Cox regression	–	Internal validation	0.812	0.753	–	Calibration curve	Nomogram

The number of candidate predictors in the models ranged from 20 to 73, with final models containing 5–20 predictors. The predictive factors were categorized into five major groups: demographic and medical history variables, physiological and laboratory indicators, scoring systems and disease severity measures, key clinical interventions, and admission-and nursing-related factors. The four most frequently included predictors were the Glasgow Coma Scale (GCS) (*n* = 9), age (*n* = 9), white blood cell count (WBC) (*n* = 8), and glucose (*n* = 7), as detailed in Table 3.

**Table 3:** Predictive factors.

Author	Candidate factors	Final number of predictors	Final predictive factors
Haoran Chen (10)	64	10	Sofa (sepsis-related organ failure assessment), minimum glucose, maximum sodium, age, mean spo2 (blood oxygen saturation), maximum temperature, maximum heart rate, minimum bun, minimum WBC, Charlson Comorbidity Index
Lingyan Fang (11)	–	14	Namely age, ethnicity type, marital status, underlying metastatic solid tumor, CCI, heart rate, GCS, WBC, glucose concentrations, sodium concentrations, potassium concentrations, MV, use of heparin and mannitol injection
Guangyong Jin (12)	–	9	marital status, type of first care unit, presence of metastatic solid tumor, first-day urine output, platelet count, mannitol administration, heparin administration, mechanical ventilation, minimum value of first-day GCS
Mariëlle K van Valburg (13)	20	8	The most important predictive factors are were age, GCS, acute physiological disturbance (as defined using APACHE-III APS; without GCS), the application of mechanical ventilation, the occurrence of acute renal failure
Jian Huang (14)	41	11	Ethnicity, age, SpO_2_, WBC, MCV, RDW, BUN, calcium, glucose, hyperlipidemia
Forhan Bin Emdad (15)	73	12	Maximum value of GCS motor response, glucose, blood urea nitrogen, GCS, white blood cells count, temperature, GCS eyes response, heart failure, services related to surgery (general but not classified) and gynecology, race, and neurologic (related to brain) surgica interventions
Jianyu Zou (16)	–	8	Age, GCS, creatinine, WBC, temperature, glucose, urine output, and bleeding volume
Longyuan Gu (17)	–	14	Gcs motor, Bicarbonate, WBC, Spo2, Heartrate, Age, NLR, Glucose, Aniongap, GCS, Rbc, Sysbp, Sodium and Gcseyes
Pedro Kurtz (18)	63	20	Mechanical ventilation, Leucocyte count, Urea, Glasgow coma scale, Creatinine, Lowest platelets, Age, Lowest mean arterial pressure, Highest temperature, Highest heart rate, Stroke type (IS, ICH, SAH), ICP monitoring, ECOG, Metastatic cancer, Altered mental status, Source of admission, Vasopres0073or requirement, Acute respiratory failure, Sex, Cardiop ylmonary arrest
Qing Mei (19)	–	5	Admission GCS, SAPS II, rebleeding, EBI, and EVD
Yuxin Wang (20)	48	16	GCS, glucose, admission age, creatinine, temperature, anion gap, respiratory rate (RR), sodium, MBP, marital status, heart rate, PT, platelets, potassium, weight, WBC
Xiao-Dan Li (21)	–	12	Age, weight, ventilation, cardiac arrhythmia, metastatic cancer, explicit sepsis, Oxford Acute Severity of Illness Score (OASIS), diastolic blood pressure (DBP), bicarbonate and chloride levels, and red blood cell (RBC) white blood cell (WBC)

Wbc, White blood cells; SpO_2_, peripheral oxygen saturation; bicarbonate; BUN, blood urea nitrogen; MCV, mean corpuscular volume; GCS, Glasgow coma scale; RDW, red blood cell distribution width; AST, aspartate aminotransferase; Polys polymorphonuclear granulocytes; NLR, neutrophil to lymphocyte ratio; Rbc, red blood cells; EBI, early brain injury; EVD, external ventricular drain; MBP, mean blood pressure; PT, Prothrombin time; OASIS, Oxford Acute Severity of Illness Score; DBP, diastolic blood pressure; ECOG, Functional impairment.

### Risk of bias and applicability assessment

**Figure 2:**
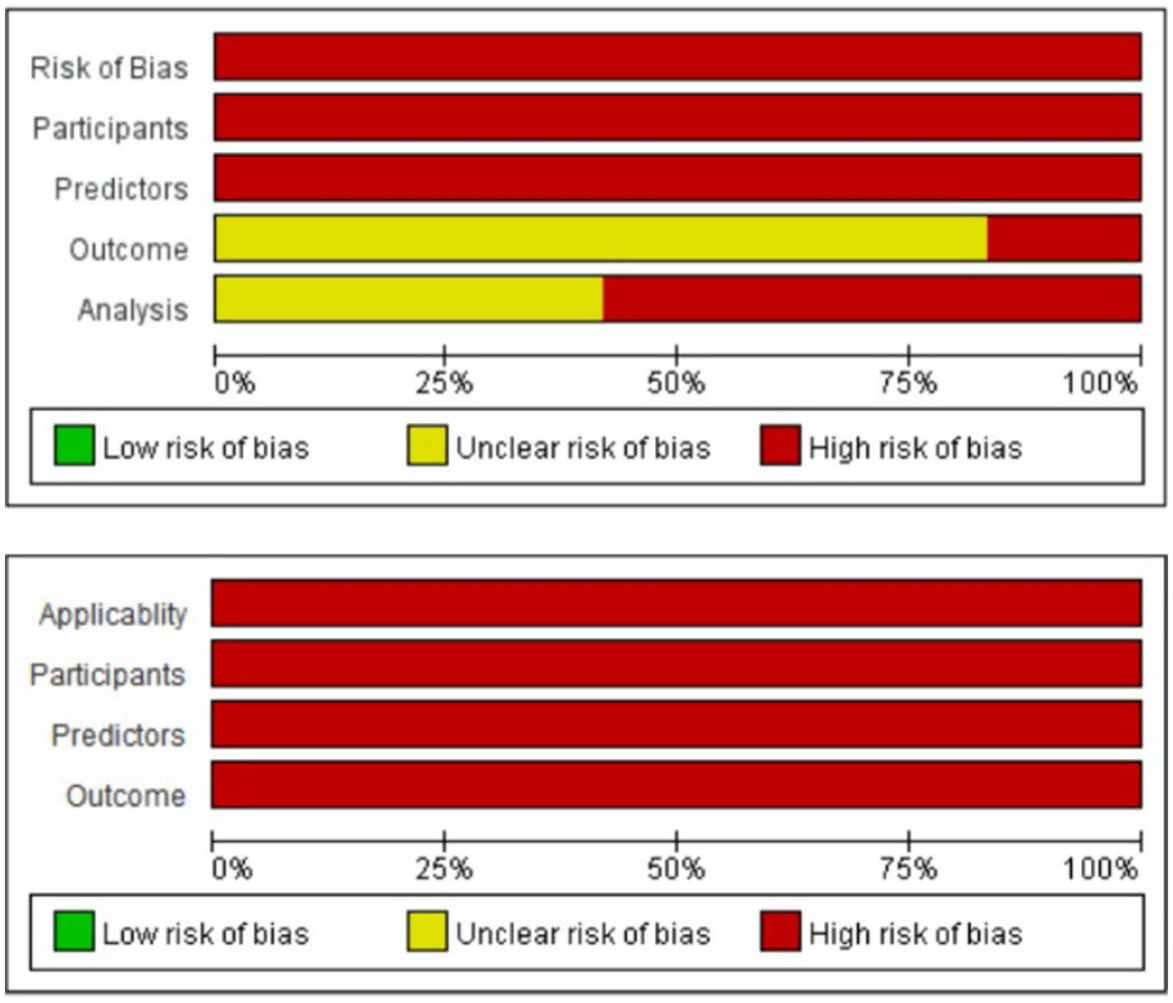
Risk of bias plot.

We assessed the risk of bias (ROB) and applicability of all included prediction models using the PROBAST tool. Our analysis revealed that all models exhibited a high risk of bias and low applicability. Detailed evaluation results are provided in Table 4, and the risk of bias is illustrated in Figure 2. Since all 12 studies employed retrospective designs and data were sourced from databases, the participant domain for all studies was rated as “high risk” (10–21). In the predictor domain, 11 studies included predictors that required subjective interpretation or were judged by different individuals with varying levels of experience (10–13, 15–21), such as the Glasgow Coma Scale (GCS) and the Charlson Comorbidity Index. Additionally, retrospective studies may involve inconsistent collection of data over time, which can impact model accuracy. Furthermore, these studies are more prone to recall bias, which could lead to the assessment of predictors with knowledge of the outcome. Therefore, all studies were rated as having a high ROB in the predictor domain. In the outcome domain, all outcome data were sourced from databases, and the use of blinding was not explicitly stated. Ten studies could not confirm whether outcomes were determined without knowledge of the predictor variables (10–12, 14–18, 20, 21). Two studies were rated as “high risk” because they included predictors directly related to the outcome definition (13, 19), the collection time of the predictors was not clearly stated in four studies (14, 16–18). Therefore, two studies were rated as high risk in the outcome domain (13, 19), while 10 studies were rated as “unclear” regarding the outcome assessment (10–12, 14–18, 20, 21). In the analysis domain, seven studies were rated as “high risk” due to sample size, the number of predictors, or improper handling of variables (10, 14, 15, 17–19, 21), while five studies were rated as “unclear” due to insufficient data handling (11–13, 16, 20). Specifically, two studies did not report the number of event outcomes used for model validation (10, 13), and six studies did not clearly specify the number of candidate predictors (11, 12, 16, 17, 19, 21). Two studies had fewer than 20 outcome events per predictor (14, 15), and one study categorized continuous variables during analysis (10). Furthermore, nine studies did not specify whether continuous and categorical predictors were appropriately handled (11–13, 15–18, 20, 21). Additionally, three studies used univariate analysis to select predictors (17, 19, 21), and one study discretized all continuous variables (10). Regarding model performance evaluation, one study employed an insufficient evaluation method and did not mention any calibration methods (15). All 12 studies were rated as having low applicability. In the participant domain, all studies were rated as having low applicability. Two studies focused on special populations: one targeting the elderly (12), and another focusing on mechanically ventilated patients with aneurysmal subarachnoid hemorrhage (aSAH) (19). The remaining 10 studies (10, 11, 13–18, 20, 21), being retrospective, were limited by data completeness, with missing key variables, and potential biases in the definition and selection of participants, resulting in low applicability. In the predictor domain, some predictors, such as the presence of metastatic solid tumors, may not be universally available in all hospitals, raising concerns about applicability (11, 12, 21). Additionally, in the remaining nine studies, retrospective data limitations led to incomplete recording of key variables at all-time points, further impacting the applicability of the predictors. In the outcome domain, since all studies rely on retrospective database data, the data sources may have certain limitations, which could restrict their external applicability.

**Table 4:** Prediction model risk of bias assessment.

Study	ROB				Applicability			Overall	
	Participants	Predictors	Outcome	Analysis	Participants	Predictors	Outcome	ROB	Applicability
Haoran Chen (10)	–	–	?	–	–	–	–	–	–
Lingyan Fang (11)	–	–	?	?	–	–	–	–	–
Guangyong Jin (12)	–	–	?	?	–	–	–	–	–
Mariëlle K van Valburg (13)	–	–	–	?	–	–	–	–	–
Jian Huang (14)	–	–	?	–	–	–	–	–	–
Forhan Bin Emdad (15)	–	–	?	–	–	–	–	–	–
Jianyu Zou (16)	–	–	?	?	–	–	–	–	–
Longyuan Gu (17)	–	–	?	–	–	–	–	–	–
Pedro Kurtz (18)	–	–	?	–	–	–	–	–	–
Qing Mei (19)	–	–	–	–	–	–	–	–	–
Yuxin Wang (20)	–	–	?	?	–	–	–	–	–
Xiao–Dan Li (21)	–	–	?	–	–	–	–	–	–

PROBAST = Prediction model Risk of Bias Assessment Tool; ROB = risk of bias. “+” indicates low ROB/low concern regarding applicability; “− “indicates high ROB/high concern regarding applicability; and “?” indicates unclear, ROB/unclear concern regarding applicability.

### Meta-analysis results

**Figure 3:**
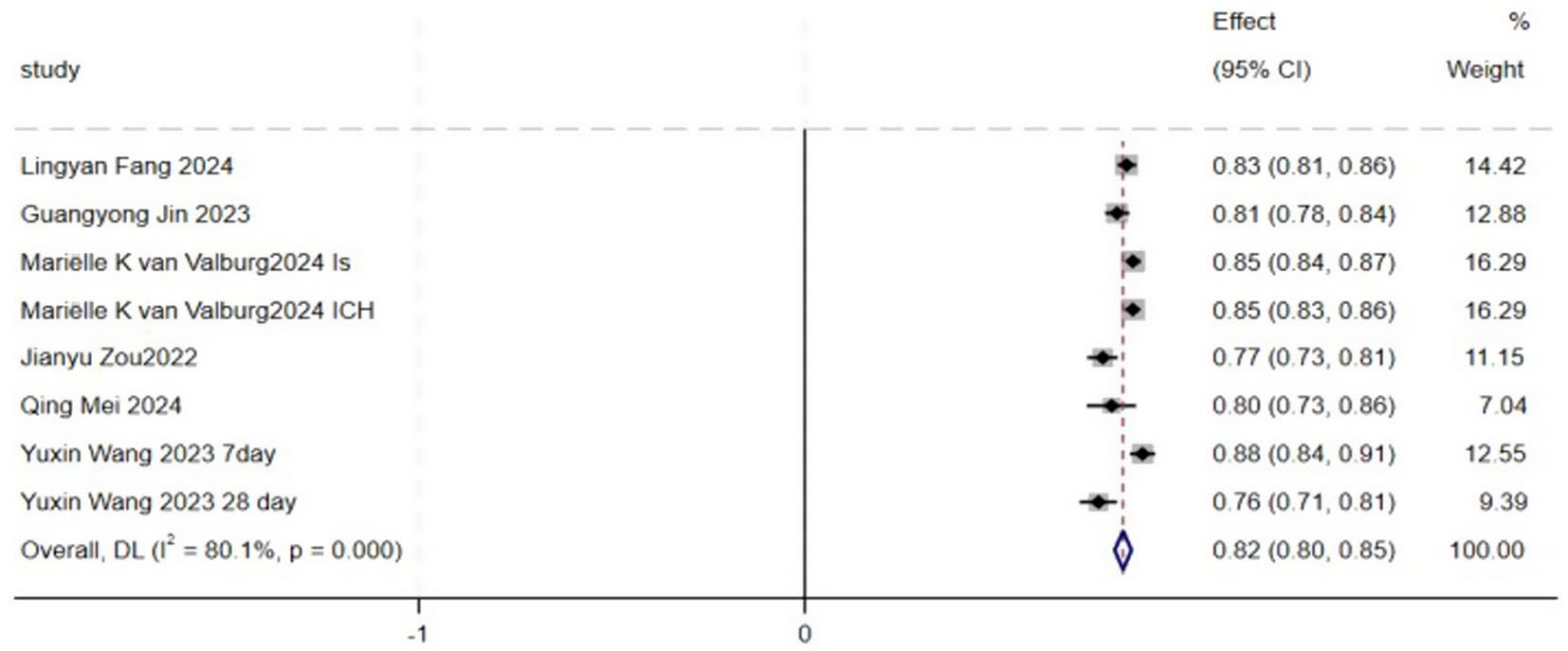
Meta-analysis.

**Figure 4:**
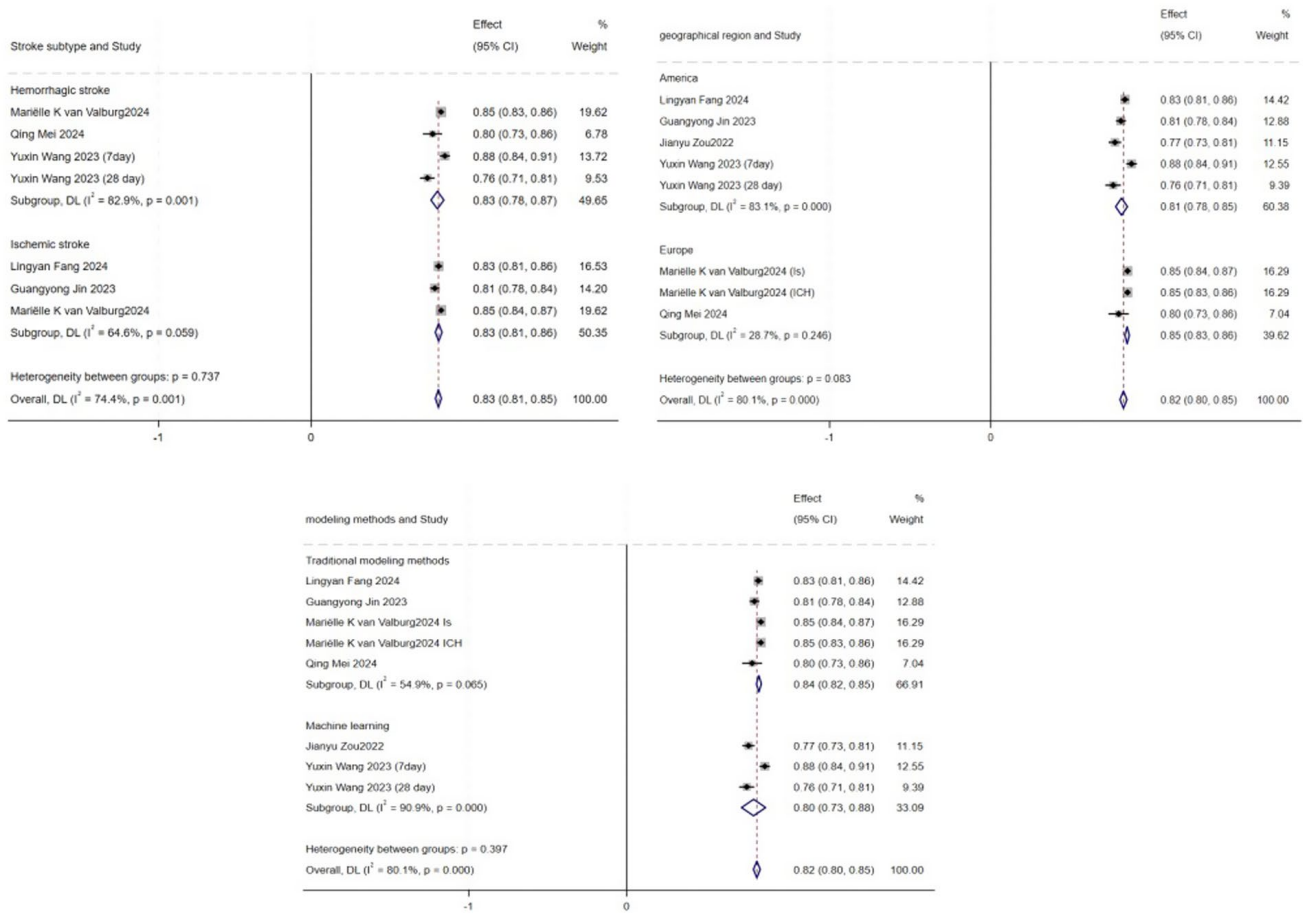
Subgroup analysis.

**Figure 5:**
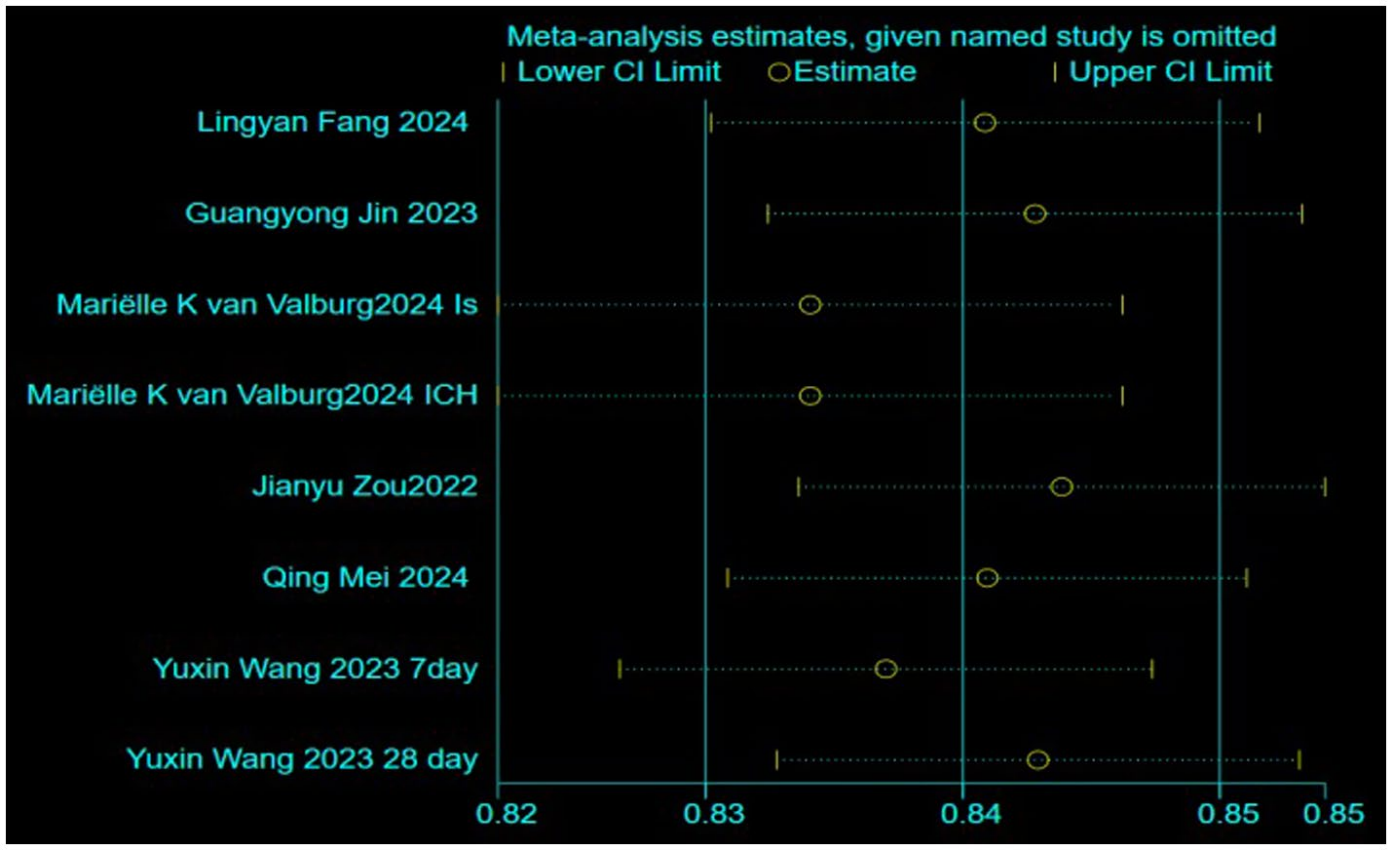
Sensitivity analysis.

Due to the lack of detailed AUC confidence interval values in some studies, only eight models from six articles were included in the meta-analysis (11–13, 16, 19, 20). The study by Mariëlle Kvan Valburg includes models for two distinct study populations (13), while Yuxin Wang’s study includes models for two different prediction time points (20), resulting in eight models from six articles. The pooled analysis showed an AUC of 0.82 (95% CI: 0.80–0.85), indicating that these models demonstrate good discriminative ability in predicting short-term mortality risk in ICU stroke patients (Figure 3). However, heterogeneity analysis revealed significant variability between the studies (I^2^ = 80.1%, *p* = 0.000). To further explore the potential sources of heterogeneity, we conducted subgroup analyses based on stroke type, modeling methods, and geographical region (Figure 4). Specifically, studies were categorized into ischemic stroke (3 models) and hemorrhagic stroke (4 models); traditional modeling methods (5 models) and machine learning methods (3 models); as well as studies conducted in America (5 models) and Europe (3 models). The subgroup analyses revealed varying degrees of heterogeneity within each subgroup. For instance, lower heterogeneity was observed in studies using traditional modeling methods (I^2^ = 54.9%) and those conducted in Europe (I^2^ = 28.7%), compared to higher heterogeneity in machine learning-based models (I^2^ = 90.9%) and studies from America (I^2^ = 83.1%). However, the between-group differences were not statistically significant (*p* > 0.05), indicating that stroke type, modeling method, and geographical region did not fully account for the observed heterogeneity. To assess the robustness of our findings, we performed a sensitivity analysis by sequentially excluding each study (Figure 5). The pooled AUC values remained stable, ranging from 0.82 to 0.85, indicating that the overall estimate is robust and reliable.

## Discussion

This systematic review included 12 studies aimed at predicting short-term mortality risk in ICU stroke patients (10–21). Most studies have reported the model’s relatively high predictive performance. These findings highlight the potential utility of these models in identifying high-risk ICU stroke patients, thereby aiding in clinical decision-making and resource allocation. The use of both traditional statistical models and machine learning techniques reflects a growing trend in the field, with the latter often outperforming traditional models in terms of predictive accuracy, as seen in models such as the Multilayer Perceptron (MLP) and Random Forest (RF), which achieved AUC values above 0.90. However, due to several factors, all models exhibited a high risk of bias. The retrospective study design is the primary factor, along with issues such as uncertainty in the number of candidate predictors, lack of clarity in handling continuous and categorical predictors, absence of calibration methods, and the inclusion of subjectively interpreted predictors. Many studies also did not specify whether outcome determination was conducted without prior knowledge of predictor variables. These issues reduce the external applicability of the models and affect the consistency of results across different settings. Additionally, most studies did not report key performance metrics such as sensitivity, specificity, and accuracy. External validation was inconsistently considered, with only a few studies conducting it (10, 14, 16, 17), while most relied solely on internal validation, potentially limiting the models’ generalizability. All 12 studies were rated as having low applicability, primarily due to their retrospective designs. Despite the high predictive performance reported, the external applicability of these models is limited. All studies relied on retrospective database data, which may introduce bias and affect the accuracy of data collection. The lack of stratified validation in comorbidity subgroups in most studies raises further concerns about their applicability to high-risk populations, despite some studies incorporating comorbidity-related predictors such as the Charlson Comorbidity Index (CCI) (11), metastatic cancer (19, 21), cardiac arrhythmia (21), and sepsis (10, 21). Meta-analysis indicating good discriminatory ability in predicting short-term mortality in ICU stroke patients. However, a high degree of heterogeneity was observed (I^2^ = 80.1%, *p* = 0.000). Subgroup analyses based on stroke type, geographical region, modeling methods did not explain the heterogeneity, which may be attributed to differences in predictor selection, study population characteristics, and sample sizes. Sensitivity analysis further confirmed the robustness of the findings, as the pooled AUC remained stable (0.82–0.85) when each study was omitted individually, suggesting that no single study unduly influenced the overall results. Due to the retrospective design, these studies have limited external validity and clinical applicability. Future research should adopt prospective designs to enhance the generalizability and clinical relevance of the models.

The short-term mortality of ICU stroke patients is influenced by several factors. Based on the results of the final modes, this study summarizes the top four risk factors as follows: GCS, WBC, age and blood glucose level. GCS is a fundamental tool for assessing consciousness in patients with central nervous system disorders, such as stroke, Systematic documentation and monitoring of GCS have become essential components of neurocritical care (22). Level of consciousness is a crucial determinant of patient outcomes, with lower GCS scores strongly associated with increased mortality risk (23). Studies have shown that a GCS score of less than 9 is a significant predictor of 30-day mortality in critically ill stroke patients (24), underscoring its importance in risk stratification and clinical decision-making. Studies have shown that inflammation is closely associated with all stages of ischemic stroke, not only contributing to the formation of ischemic injury but also exacerbating neurological deterioration (25, 26). Leukocytosis alone has been demonstrated to correlate with neurological deterioration in patients with acute ischemic stroke and is associated with worse outcomes (27). Research has found that a white blood cell (WBC) count >12.5 upon hospital admission is linked to an increased 30-day mortality rate. Additionally, a reduction in leukocyte counts by more than 3 Gpt/L from admission to the third day was associated with higher mortality, whereas stable leukocyte levels (ranging from −3 to +3 Gpt/L) were associated with a reduced risk of 30-day mortality following intracerebral hemorrhage (ICH) (28). These findings underscore the importance of leukocyte count as a key factor in mortality prediction in stroke patients, particularly those with ICH, as leukocytes also play a critical role in secondary brain injury following ICH (29). Age is a critical, non-modifiable risk factor for ischemic stroke (30). The aging process has a significant impact on the pathophysiology of stroke, increasing both the risk of occurrence and the severity of functional outcomes, and it affects neuronal activity and viability, glial cell function, the structure and function of cerebral blood vessels and the blood–brain barrier (BBB), which can make blood vessels more prone to rupture, thereby increasing the risk of hemorrhagic transformation following ischemic stroke (31–34). Some studies have shown that older individuals tend to experience higher stroke-related mortality and poorer post-stroke quality of life (35, 36). Our analysis confirms that age is a critical risk factor in predictive models, emphasizing its role in mortality risk assessment. Diabetes mellitus is a significant risk factor for stroke incidence, recurrence and mortality, it contributing to over one-fifth of stroke-related deaths (37–41). Patients with both stroke and diabetes exhibit substantially higher mortality rates (42). Hyperglycemia during the acute phase of stroke is closely linked to poor outcomes. Blood glucose levels ≥155 mg/dL within the first 24–72 h post-stroke are associated with greater glycemic variability, increased complications, and higher three-month mortality (43). In ischemic stroke, hyperglycemia is strongly correlated with larger infarcts, worse functional outcomes, and increased mortality (44). Sustained hyperglycemia following stroke accelerates brain injury (45). These findings emphasize the clinical importance of GCS, WBC, age, and blood glucose levels in predicting short-term mortality in ICU stroke patients. Incorporating these variables in future predictive models could improve accuracy and clinical utility, ensuring more effective patient management.

### Limitations

(1) Due to the lack of AUC values for internal and external validation, we used the AUC values from model construction for the meta-analysis. (2) The lack of AUC confidence interval values for some models resulted in the inclusion of only 8 models from 6 studies, potentially affecting the meta-analysis results. (3) The absence of key performance metrics such as sensitivity, specificity, and accuracy limited the comprehensiveness of the meta-analysis. (4) Only English-language studies were included, which may have excluded relevant research published in other languages, potentially affecting the comprehensiveness of the results.

## Conclusion

This study demonstrates that prediction models for short-term mortality in ICU stroke patients exhibit good to excellent discriminatory performance. However, due to high risk of bias and low applicability, the overall quality of these models remains suboptimal. Our analysis identifies GCS, WBC, age, and blood glucose levels as the most frequently identified and important predictors of short-term mortality. Incorporating these factors into future models can significantly enhance their predictive accuracy and clinical relevance. Future research, guided by the PROBAST tool, should adopt rigorous methodologies with prospective designs and conduct large-scale, multicenter, externally validated studies to improve both the clinical applicability and reliability of these models.

## Data Availability

The original contributions presented in the study are included in the article/Supplementary material, further inquiries can be directed to the corresponding authors.
