# Supplementary material for: Risk prediction models for short-term mortality in ICU stroke patients: a systematic review and meta-analysis
Source: Front Neurol. 2025 Jul 16;16:1623645. doi: 10.3389/fneur.2025.1623645 (PMC12307426; doi:10.3389/fneur.2025.1623645)
Supplement: Supplementary file 1 [file Table_1.DOCX]

Search terms for PubMed

| #1 | (((((((((Stroke[MeSH Terms]) OR (Strokes[Title/Abstract])) OR (Cerebrovascular Accident[Title/Abstract])) OR (Cerebral Stroke[Title/Abstract])) OR (Stroke, Cerebra[Title/Abstract])) OR (Cerebrovascular Apoplexy[Title/Abstract])) OR (Brain Vascular Accident[Title/Abstract])) OR (Cerebrovascular Stroker[Title/Abstract])) OR (Apoplexy[Title/Abstract])) OR (CVA[Title/Abstract]) | 216,087 |
| --- | --- | --- |
| #2 | ((((Mortality[MeSH Terms]) OR (Mortalities[MeSH Terms])) OR (Mortality Rate[MeSH Terms])) OR (Death Rate[MeSH Terms])) OR (Differential Mortality[MeSH Terms]) | 438,820 |
| #3 | ((((((((risk prediction[Title/Abstract]) OR (risk score[Title/Abstract])) OR (Risk calculation[Title/Abstract])) OR (risk assessment[Title/Abstract])) OR (AUC[Title/Abstract])) OR (roc curve[Title/Abstract])) OR (area under the curve[Title/Abstract])) OR (Nomogram[Title/Abstract])) OR (prediction model[Title/Abstract]) | 407,744 |
| #4 | #1 AND #2 AND #3 AND | 250 |

Search terms for Embase

| #1 | 'cerebrovascular accident'/exp OR 'cerebrovascular accident' OR strokes:ab,ti OR 'cerebral stroke':ab,ti OR 'stroke, cerebral':ab,ti OR 'cerebrovascular apoplexy':ab,ti OR 'brain vascular accident':ab,ti OR 'cerebrovascular stroker':ab,ti OR apoplexy:ab,ti OR cva:ab,ti | 512,592 |
| --- | --- | --- |
| #2 | 'risk prediction'/exp OR 'risk prediction' OR 'risk score':ab,ti OR 'risk calculation':ab,ti OR 'risk assessment':ab,ti OR auc:ab,ti OR nomogram:ab,ti OR 'prediction model':ab,ti OR 'roc curve':ab,ti OR 'area under the curve':ab,ti | 572,262 |
| #3 | 'mortality'/exp OR mortality OR mortalities:ab,ti OR 'mortality rate':ab,ti OR 'death rate':ab,ti OR 'differential mortality':ab,ti | 2,184,731 |
| #4 | #1 AND #2 AND #3 | 2611 |

Search terms for Web of Science

| #1 | Stroke (Abstract) or Strokes (Abstract) or Cerebrovascular Accident (Abstract) or Cerebral Stroke (Abstract) or Stroke, Cerebral (Abstract) or Cerebrovascular Apoplexy (Abstract) or Brain Vascular Accident (Abstract) or Cerebrovascular strokes (Abstract) or Apoplexy (Abstract) or CVA (Abstract) | 71,146 |
| --- | --- | --- |
| #2 | prediction model (Abstract) OR Nomogram (Abstract) OR area under the curve (Abstract) OR roc curve (Abstract) OR AUC (Abstract) OR risk assessment (Abstract) OR Risk calculation (Abstract) OR risk score (Abstract) OR risk prediction (Abstract) | 40,467 |
| #3 | Differential Mortality (Abstract) OR Death Rate (Abstract) OR Mortality Rate (Abstract) OR Mortalities (Abstract) OR Mortality (Abstract) | 56,979 |
| #4 | #1 AND #2 AND #3 | 261 |

Search terms for Cochrane

| #1 | [Stroke] explode all trees or (Strokes):ab OR (Cerebrovascular Accident or Brain Vascular Accident or Cerebrovascular Stroker or Apoplexy or CVA):ab OR (Cerebral Stroke):ab OR (Stroke, Cerebral):ab OR (Cerebrovascular Apoplexy):ab (Word variations have been searched) | 58638 |
| --- | --- | --- |
| #2 | (risk prediction):ab OR (risk score):ab OR (Risk calculation):ab OR (risk assessment):ab OR (AUC):ab or (roc curve):ab OR (area under the curve):ab OR (Nomogram):ab OR (prediction model):ab | 203985 |
| #3 | [Mortality] explode all trees or (Mortalities):ab OR (Mortality Rate):ab OR (Death Rate):ab OR (Differential Mortality):ab | 130947 |
| #4 | #1 AND #2 AND #3 | 3752 |
